# Identification of proteins associated with pyrethroid resistance by iTRAQ-based quantitative proteomic analysis in *Culex pipiens pallens*

**DOI:** 10.1186/s13071-015-0709-5

**Published:** 2015-02-10

**Authors:** Weijie Wang, Yuan Lv, Fujin Fang, Shanchao Hong, Qin Guo, Shengli Hu, Feifei Zou, Linna Shi, Zhentao Lei, Kai Ma, Dan Zhou, Donghui Zhang, Yan Sun, Lei Ma, Bo Shen, Changliang Zhu

**Affiliations:** Department of Pathogen Biology, Nanjing Medical University, Nanjing, China; Jiangsu Province Key Laboratory of Modern Pathogen Biology, Nanjing Medical University, Nanjing, China; Department of Pathogen Biology, Hebei Medical University, Shijiazhuang, China

**Keywords:** Insecticide resistance, Pyrethroids, Proteomic, iTRAQ, P450, *Culex pipiens pallens*

## Abstract

**Background:**

Mosquito control based on chemical insecticides is considered as an important element in the current global strategies for the control of mosquito-borne diseases. Unfortunately, the development of pyrethroid resistance in important vector mosquito species jeopardizes the effectiveness of insecticide-based mosquito control. To date, the mechanisms of pyrethroid resistance are still unclear. Recent advances in proteomic techniques can facilitate to identify pyrethroid resistance-associated proteins at a large-scale for improving our understanding of resistance mechanisms, and more importantly, for seeking some genetic markers used for monitoring and predicting the development of resistance.

**Methods:**

We performed a quantitative proteomic analysis between a deltamethrin-susceptible strain and a deltamethrin-resistant strain of laboratory population of *Culex pipiens pallens* using isobaric tags for relative and absolute quantitation (iTRAQ) analysis. Gene Ontology (GO) analysis was used to find the relative processes that these differentially expressed proteins were involved in. One differentially expressed protein was chosen to confirm by Western blot in the laboratory and field populations of *Cx. pipiens pallens*.

**Results:**

We identified 30 differentially expressed proteins assigned into 10 different categories, including oxidoreductase activity, transporter activity, catalytic activity, structural constituent of cuticle and hypothetical proteins. GO analysis revealed that 25 proteins were sub-categorized into 35 hierarchically-structured GO classifications. Western blot results showed that CYP6AA9 as one of the up-regulated proteins was confirmed to be overexpressed in the deltamethrin-resistant strains compared with the deltamethrin-susceptible strains both in the laboratory and field populations.

**Conclusions:**

This is the first study to use modern proteomic tools for identifying pyrethroid resistance-related proteins in *Cx. pipiens*. The present study brought to light many proteins that were not previously thought to be associated with pyrethroid resistance, which further expands our understanding of pyrethroid resistance mechanisms. CYP6AA9 was overexpressed in the deltamethrin-resistant strains, indicating that CYP6AA9 may be involved in pyrethroid resistance and may be used as a potential genetic marker to monitor and predict the pyrethroid resistance level of field populations.

**Electronic supplementary material:**

The online version of this article (doi:10.1186/s13071-015-0709-5) contains supplementary material, which is available to authorized users.

## Background

Mosquito-borne diseases, such as malaria, dengue fever, yellow fever, filariasis, West Nile fever, constitute a major burden for public health problems worldwide [1-4]. Mosquito control is considered as an important element in the current global strategies for the control of major mosquito-borne diseases [4,5]. Since the advent of dichlorodiphenyltrichloroethane (DDT) and other organochlorine insecticides in the 1940s, the control of mosquito populations is often based on chemical insecticides, and their use has been shown to prevent transmission of disease pathogens to humans [2]. In general four different chemical classes of synthetic insecticides that target adult mosquitoes are sequentially used historically: organochlorines, organophosphates, carbamates and pyrethroids. Pyrethroids such as deltamethrin are particularly important and now widely applied due to several advantages over other insecticides in term of low cost, safety (less toxic to mammals) and duration of residual action [6]. Unfortunately, long-term intensive and widespread use of pyrethroids has led to the development of pyrethroid resistance in important vector mosquito species, making pyrethroid use ineffective and limiting the available options for disease control [7]. Consequently, pyrethroid resistance has become a global problem, and been considered a serious public health challenge [1,5,7,8]. It is an urgent need to develop a strategy of pyrethroid resistance management, and a comprehensive knowledge of pyrethroid resistance mechanisms is considered fundamental to this strategy. Generally, three types of resistance mechanisms have been described [7,9-12]: metabolic resistance (alterations in the levels or activities of detoxification enzymes), target site resistance (mutations in the sodium channel) and cuticular resistance (modifications in the insect cuticle and/or digestive tract for preventing or slowing down the absorption or penetration of pyrethroids).

Pyrethroid resistance is a complicated and polygenic inheritance phenomenon [13]. Identification of genes associated with pyrethroid resistance is conducive to clarify the resistance mechanisms. To date, dramatic progress has been made in identifying pyrethroid resistance-associated genes at the transcriptional level by suppression subtractive hybridization (SSH) [14], cDNA microarray [15,16], RNA-seq [17,18], etc. Consequently, numerous genes associated with pyrethroid resistance have been observed, including cytochrome P450s (CYPs or P450s) [9,19,20], glutathione S-transferases (GSTs) [21], ribosomal proteins [22-24], and others. However, protein is the executor to perform the gene function. Protein functions that depend on post-translational and protein-protein interactions cannot be predicted through transcriptional level analysis. More importantly, the relationships between mRNA transcript and protein abundances were shown to be only partially correlated with a squared Pearson correlation coefficient of ~0.40 [25]. So, it is significant to identified pyrethroid resistance-associated proteins. Proteomics have been emerged as a powerful method to investigate protein changes at the cellular level. Traditionally, proteomics based on two-dimensional gel electrophoresis (2-DE) have been applied to identify differentially expressed proteins, including the identification of proteins associated with insecticide resistance in mosquitoes [26,27] and other insect species [28,29]. However, there are some disadvantages of 2-DE such as difficulties resolving hydrophobic proteins and low abundance proteins with extreme pI and molecular weights [30]. With the development of modern biotechnology, a series of new techniques in the field of proteomics have emerged, especially in the availability of highly sensitive proteomic platforms, such as isobaric tag for relative and absolute quantitation (iTRAQ), which is beneficial to obtain information not accessible using 2-DE. The technique iTRAQ has been considered as a superior choice due to its high proteome coverage and labeling efficiency [31], and widely employed in quantitative proteomics.

In the current study, we intended to carry out a quantitative proteomic analysis between a deltamethrin-susceptible strain and a deltamethrin-resistant strain in the laboratory population of *Cx. pipiens pallens* using iTRAQ labeling coupled with liquid chromatography/tandem mass spectrometric (LC-MS/MS) analysis. Differentially expressed proteins were identified and some were further validated in the laboratory and field mosquito populations by Western blot. Finally, one interesting protein CYP6AA9 was confirmed to be up-regulated in the deltamethrin-resistant strain compared with the deltamethrin-susceptible strain in the laboratory and field mosquito populations, which indicated that CYP6AA9 was involved in pyrethroid resistance and may be used as a potential genetic marker to monitor and predict the pyrethroid resistance level of field mosquito populations.

## Methods

### Ethics statement

No specific permits were required for the field studies. All field mosquito populations were collected from public areas. No sites were protected by law and this study did not involve endangered or protected species.

### Mosquito strains and insecticide susceptibility tests

Two laboratory-reared strains of *Cx. pipiens pallens* were used in this study: a deltamethrin-susceptible (Lab-DS) strain and a deltamethrin-resistant (Lab-DR) strain. The Lab-DS strain originated from Tangkou Village (Shandong Province), and has been colonized in the insectary without exposure to any insecticides since 2009. The Lab-DS strain was repeatedly selected for 32 generations at the larval stage with deltamethrin at the median lethal concentration (LC_50_), and the deltamethrin-selected strain was defined as the Lab-DR strain. A detailed selection procedure was described previously [32,33]. The LC_50_ of the Lab-DS strain and the Lab-DR strain was 0.03 and 0.85 mg/L, respectively.

In addition, three field populations of *Cx. pipiens pallens* were also used: BB (Bengbu City, Anhui Province), JN (Jining City, Shandong Province) and NJ (Nanjing City, Jiangsu Province) population. BB population was collected in July 2012; JN population and NJ population were collected in August 2013. For each field mosquito population, larvae were collected in public areas of the respective location, and were brought back to the insectary after morphological identification. Non-blood-fed female mosquitoes of the three field populations aged 1–3 days post emergence were exposed to discriminating doses of deltamethrin (0.05%) for susceptibility tests using deltamethrin-impregnated papers, as described by the standard WHO testing protocol [6,34]. The deltamethrin-impregnated papers were obtained from University Sains Malaysia (Penang, Malaysia). At least four replicates were done for each field population. The controls were exposed to control papers impregnated with silicone oil. After exposure for 1 hour, mosquitoes were transferred to recovery cups. If knocked down rate was less than 80%, knock-down was monitored for a further 20 min in the recovery cups [6]. Then the number of knocked-down mosquitoes was recorded. Based on previous studies [35,36], the knocked-down mosquitoes were defined as deltamethrin-susceptible (DS) strains and preserved in Eppendorf tubes without following recovery period for overcoming post-mortem protein degradation. Meanwhile, the mosquitoes that were not knocked down were maintained on 10% sugar solution for 24 hours. At the end of recovery period, the surviving mosquitoes were defined as deltamethrin-resistant (DR) strains. All the mosquito samples were preserved for further analysis at −80°C.

All the mosquito populations were reared at a constant room temperature of approximately 28°C and 75% relative humidity with a photophase of 14 h and a scotophase of 10 h. Adult mosquitoes were provided with 10% sugar solution.

### Protein preparation for iTRAQ experiments

Total proteins of each sample were extracted separately from whole bodies of 20 fourth-instar larvae, 20 pupae, 20 adult males (2–3 days old) and 20 adult females (without blood feeding, 2–3 days old) of the Lab-DS and Lab-DR strains. Larvae and pupae were rinsed 3 times using deionized distilled water (ddH_2_O) to remove food particles and molted skin. Briefly, the procedure for protein extraction was as follows. Each sample was placed in pre-cooled Eppendorf tube and homogenized thoroughly with a Pellet Pestle Motor (Kontes, USA) in a solution of cold acetone containing 10% trichloroacetic acid (TCA) and 10 mM dithiothreitol (DTT). After homogenization, each sample was kept overnight at −20°C. Then protein pellets were collected by centrifuging and resuspended in lysis buffer (7 M urea, 2 M thiourea, 4% CHAPS), containing 1 mM phenylmethylsulfonyl fluoride (PMSF), 2 mM ethylenediaminetetraacetic acid (EDTA) and 10 mM DTT. Samples were sonicated and centrifuged, and then the supernatant was reduced and alkylated by 10 mM DTT and 55 mM iodoacetamide (IAA). The treated proteins were precipitated with chilled acetone (1:4) at −20°C overnight. The precipitants were resuspended in 500 mM triethylammonium bicarbonate (TEAB), then sonicated and centrifuged. Finally, the protein content of the supernatant was determined using a Bradford Protein Assay Kit (CWBIO, China). Bovine serum albumin (BSA) was used as the standard.

### Isobaric labeling

According the protein concentration of each sample, 25 *μ*g of proteins from each life stage were combined to form a protein pool for the Lab-DS and Lab-DR strains. The protein pool of each strain was digested with MS-grade trypsin gold (Promega, USA) with the ratio of protein: trypsin = 30: 1 at 37°C for 16 h. After trypsin digestion, peptides were labeled with 8-plex iTRAQ (Applied Biosystems, USA) following the manufacturer’s protocol. The iTRAQ regents were used to label the tryptic peptides as follows: iTRAQ tag 113 and 121 for the Lab-DS strain; iTRAQ tag 115 and 119 for the Lab-DR strain.

The labeled peptides were pooled, eluted and resolved into 10 fractions using Ultremex SCX column containing 5-*μ*m particles (Phenomenex, USA). The eluted fractions were desalted using a Strata X C18 column (Phenomenex, USA) and dried under vacuum.

### Nano LC − MS/MS analysis

Each fraction was resuspended in certain volume of buffer A (2% ACN, 0.1% FA) and centrifuged at 20,000 × g for 10 min. The final concentration of peptide was about 0.5 *μ*g/*μ*l on average in each fraction. Supernatant was loaded on an Shimadzu LC-20 AD nanoHPLC using the autosampler. The peptides were subjected to nanoelectrospray ionization followed by tandem mass spectrometry (MS/MS) in an LTQ Orbitrap Velos (Thermo Fisher Science, USA) coupled online to the HPLC. Intact peptides were detected in the Orbitrap at a resolution of 60,000. Peptides were selected for MS/MS using high energy collision dissociation (HCD) operating mode with a normalized collision energy setting of 45%. Ion fragments were detected in the LTQ. A data-dependent procedure that alternated between one MS scan followed by eight MS/MS scans was applied for the eight most abundant precursor ions above a threshold ion count of 5,000 in the MS survey scan. The electrospray voltage applied was 1,500 V. Automatic gain control (AGC) was used to prevent overfilling of the ion trap; 1 × 10^4^ ions were accumulated in the ion trap for generation of HCD spectra. For MS scans, the *m/z* scan range was 350 to 2,000 Da.

### Data processing, protein Identification and statistical analyses

The resulting MS/MS spectra were searched against the composite database of *Cx. quinquefasciatus* (CpipJ1.2 dataset) available at VectorBase database (http://www.vectorbase.org/) [37] using the Mascot software (version 2.3.02, Matrix Science, UK). For protein identification and quantification, a peptide mass tolerance of 10 ppm was allowed for intact peptide masses and 0.05 Da for fragmented ions. One missed cleavage was allowed in the trypsin digests; carbamidomethylation of cysteine was considered a fixed modification, and the conversion of N-terminal glutamine to pyroglutamic acid and methionine oxidation were considered variable modifications. All identified peptides had an ion score above the Mascot peptide identity threshold, and a protein was considered identified if at least one such unique peptide match was apparent for the protein. For protein-abundance ratios measured using iTRAQ, we took a 1.2-fold change as the threshold and a two-tailed *p* < 0.05 to identify significant changes.

### Functional enrichment analyses

The logic algorithm for set operations was applied to further screen for differentially expressed proteins identified in present study. Gene Ontology (GO) functional annotation was carried out using Blast2GO software, an integrated GO annotation and data mining tool [38], and GO enrichment analysis was performed to provide all GO terms that were significantly enriched in differentially expressed proteins.

### Antibody preparation

CYP6AA9, one of differentially expressed proteins, was chosen to be validated further by Western blot. However, there were no commercial antibodies available, thus we prepared the rabbit polyclonal antibodies against it. The anti-peptide approach to antibody production overcomes the need to use purified P450s as antigens and is considered as a relatively simple, rapid, and effective method of producing antibodies [39]. Moreover, a high degree of binding specificity can be achieved by directing anti-peptide antibody toward unique regions of CYP6AA9. Therefore, we firstly designed a peptide for CYP6AA9 according to the amino sequence of CYP6AA9 of *Cx. quinquefasciatus* (VectorBase ID: CPIJ800196). The peptide sequence is NH_2_-DPDIYPNPSQFDPDRC-CONH_2_, which was also confirmed to be exist in *Cx. pipiens pallens* by PCR. The peptide synthesis and polyclonal antibody production were carried out by Abgent (Suzhou, China).

### Validation of CYP6AA9 expression in the laboratory population by Western blot

Western blot analyses were performed to confirm the expression of CYP6AA9 at different life stages in the laboratory population. Total proteins of each sample were extracted separately from whole bodies of 10 fourth-instar larvae, 10 pupae, 10 adult males (2–3 days old) and 10 adult females (without blood feeding, 2–3 days old) from the Lab-DS and Lab-DR strains. Each sample was homogenized thoroughly with a Pellet Pestle Motor (Kontes, USA) in lysis buffer containing 200 *μ*l RIPA (Beyotime, China) and 1 mM PMSF. Then samples were ultrasonicated for 30–60 second on ice bath, and centrifuged for 10 min at 15,000 x g at 4°C. Protein concentrations were determined with the enhanced BCA protein assay kit (Beyotime, China). Equal amount proteins (80 *μ*g) of different samples were separated on a 10% SDS-PAGE gel with a Tris-glycine running buffer (25 mM Tris-base, 250 mM glycine, 0.1% SDS, pH 8.3) and blotted onto nitrocellulose membranes (Millipore, USA) in blotting buffer (25 mM Tris-base, 192 mM glycine and 20% methanol, pH 8.3) for 1 h at 300 mA. The blotted membranes were then blocked in 5% skimmed milk in TBST (20 mM Tris (pH 8.0), 150 mM NaCl, 0.05% Tween-20) for 1 h and washed with TBST. Subsequently, the membranes were incubated with anti-CYP6AA9 (1:500 dilution) or anti-β-actin (1:2,000 dilution, Abgent, China) antibodies in TBST at 4°C overnight, and washed with TBST. After treating with horseradish peroxidase-conjugated secondary antibodies (1:3,000 dilution) in TBST for 2 h, the membranes were washed with TBST. At last, the bound antibodies were recognized by using SuperSignal® West Pico Chemiluminescent Substrate Kit (Thermo Fisher Science, USA), and the signals were detected using a Bio-Rad ChemiDoc XRS scanner and Quantity One software (Bio-Rad, USA). β-actin was used as internal control.

### Validation of CYP6AA9 expression in the field populations by Western blot

After insecticide susceptibility tests for the three field populations, each population was discriminated into two phenotypes: DS strain and DR strain. We extracted separately the total proteins from the BB-DS, BB-DR, JN-DS, JN-DR and NJ-DR strains, and compared the expression of CYP6AA9 in different strains of each population by Western blot. Meanwhile, the Lab-DS strain was taken as control group. The procedure of Western blot was described above. However, the expression of CYP6AA9 in the NJ-DS strain was not determined due to the lack of enough samples for comparison.

## Results

### Quantitative proteomic analysis by iTRAQ labeling

**Figure 1:**
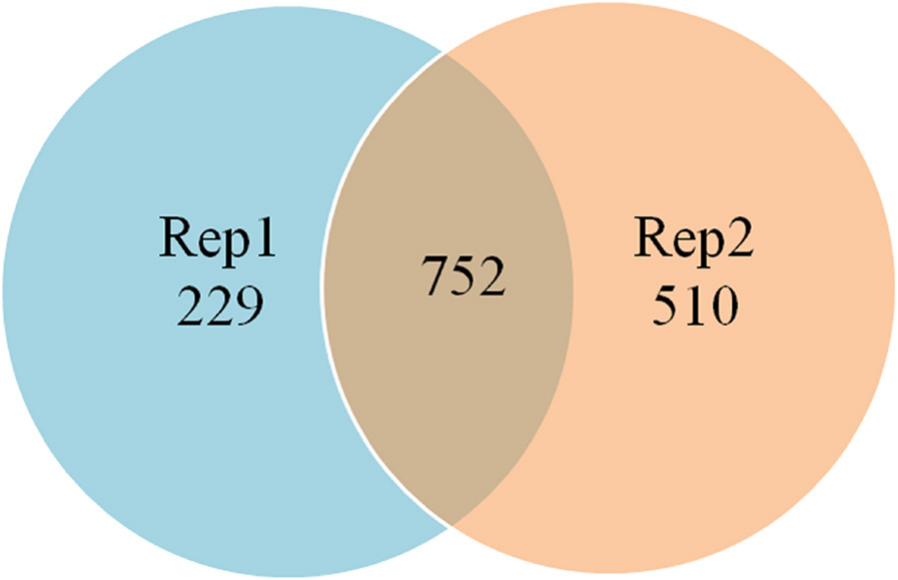
**Venn diagram of proteins identified in both biological replicates.**

In this study, two independent biological replicates (Rep1 and Rep2 experiments) were successively performed. The total number of spectra detected in *Cx. pipiens pallens* proteins was 72,235 and 65,613, respectively (Table 1). Finally, a total of 1,491 proteins were identified, of which 752 proteins were found in both replicates (Figure 1, Additional file 1: Table S1). According to the criteria for defining differentially expressed proteins (fold change ratio > 1.2 and *p* < 0.05), 90 up-regulated and 86 down-regulated proteins were identified in the Lab-DR strain comparing with the Lab-DS strain in the Rep1 experiment (Table 2, Additional file 2: Table S2), and 77 up-regulated and 89 down-regulated proteins in the Rep2 experiment (Table 2, Additional file 3: Table S3). Taking into the results of the Rep1 and Rep2 experiments, there were 30 differentially expressed proteins identified in both replicates (Table 2), including 15 up-regulated and 15 down-regulated proteins (Tables 3 and 4). In addition, one representative MS/MS spectrum of CYP6AA9 was shown (Additional file 4: Figure S1)

**Table 1:** **Summary of iTRAQ metrics from** ***Cx. pipiens pallens*** **proteome**

**Metrics**	**Rep1**	**Rep2**
Total spectra	72235	65613
Spectra	6132	8499
Unique specra	5353	7617
Peptide	2323	3552
Unique peptide	2188	3342
Protein	981	1262

**Table 2:** **The number of differentially expressed proteins identified in the Rep1 and Rep2 experiments**

	**Up-regulated**	**Down-regulated**	**Total**
**Rep1**	90	86	176
**Rep2**	77	89	166
**Rep1& Rep2**	15	15	30

**Table 3:** List **of up-regulated proteins in the Lab-DR strain comparing with the Lab-DS strain**

**Protein name**	**VectorBase ID**	**Protein coverage (%)**		**NO.peptide**		**Fold changes**		**Function**
		**Rep1**	**Rep2**	**Rep1**	**Rep2**	**Rep1**	**Rep2**	
CYP6AA9	CPIJ800196	7.4	5.8	3	2	1.974	2.439	Oxidoreductase activity
NADH dehydrogenase iron-sulfur protein 3, mitochondrial precursor	CPIJ013503	7.7	10.2	6	4	1.278	1.453	Oxidoreductase activity
ATP synthase B chain, mitochondrial precursor	CPIJ006067	10.9	26.9	2	6	1.459	1.556	Transporter activity
Hydrogen-transporting ATP synthase, G-subunit, putative	CPIJ801865	14.9	23.8	1	2	1.403	1.559	Transporter activity
CRAL/TRIO domain-containing protein, putative	CPIJ009578	15.6	7.1	4	2	1.493	1.624	Transporter activity
Cuticle protein CP14.6 precursor	CPIJ802440	11.5	11.5	1	1	1.774	3.561	Structural constituent of cuticle
Trehalose-6-phosphate synthase	CPIJ801662	8.7	5.9	6	4	1.772	1.426	Catalytic activity
utp-glucose-1-phosphate uridylyltransferase 2	CPIJ006068	2.5	14	1	6	1.621	1.331	Transferase activity
galactokinase	CPIJ015186	5.5	2.9	2	1	1.207	1.206	Transferase activity;
DEAD box ATP-dependent RNA helicase	CPIJ801619	11.6	5.7	4	2	1.216	1.307	Hydrolase activity
Histone cluster 1, putative	CPIJ012406	28.2	35.9	3	4	1.236	1.297	DNA binding;
T-complex protein 1 subunit epsilon	CPIJ801444	3.9	6.3	2	3	1.242	1.518	Nucleic acid binding
NADH-ubiquinone oxidoreductase 42 kda subunit	CPIJ011693	11.9	16.8	4	5	1.284	1.218	Phosphotransferase activity
Conserved hypothetical protein (prag01)	CPIJ017479	36	36	2	2	1.337	1.635	
Conserved hypothetical protein	CPIJ009715	12.4	16.6	3	4	1.799	1.983

**Table 4:** **List of down-regulated proteins in the Lab-DR strain comparing with the Lab-DS strain**

		**Protein coverage (%)**		**NO.peptide**		**Fold changes**		
**Protein name**	**VectorBase ID**	**Rep1**	**Rep2**	**Rep1**	**Rep2**	**Rep1**	**Rep2**	**Function**
Anamorsin, putative	CPIJ801647	4.6	4.6	1	1	0.524	0.545	Electron carrier activity
Cuticle protein, putative	CPIJ001836	26.5	51	3	5	0.57	0.729	Structural constituent of cuticle
Pupal cuticle protein 78E, putative	CPIJ012466	6	23.9	1	5	0.635	0.722	Structural constituent of cuticle
Odorant binding protein OBP43	CPIJ017326	6.5	38.4	1	3	0.675	0.649	Odorant binding
Thymosin isoform 1	CPIJ012935	10	32.3	1	3	0.682	0.691	Actin binding
Serine/arginine rich splicing factor	CPIJ801552	6.5	14.5	1	3	0.809	0.804	Nucleic acid binding
Calponin/transgelin	CPIJ801665	28	26.9	3	3	0.823	0.768	Protein binding
Sensory appendage protein, putative	CPIJ801977	8.8	19.2	1	2	0.656	0.788	
Conserved hypothetical protein (CPR135)	CPIJ014778	9.2	12.7	2	3	0.664	0.832	Structural constituent of cuticle
Hypothetical protein (CPR110)	CPIJ009316	8.6	21.1	1	4	0.453	0.82	Structural constituent of cuticle
Conserved hypothetical protein	CPIJ801684	8.2	7.8	10	10	0.809	0.773	Lipid transporter activity
Conserved hypothetical protein	CPIJ002809	9.6	3.2	2	1	0.776	0.738	Chaperone binding
Conserved hypothetical protein	CPIJ011923	19.3	24.1	2	3	0.709	0.756	
Hypothetical protein	CPIJ000341	11.4	14.2	2	3	0.464	0.75	
Hypothetical protein	CPIJ006085	4.7	4.7	1	1	0.512	0.747	

.

### GO analysis

**Figure 2:**
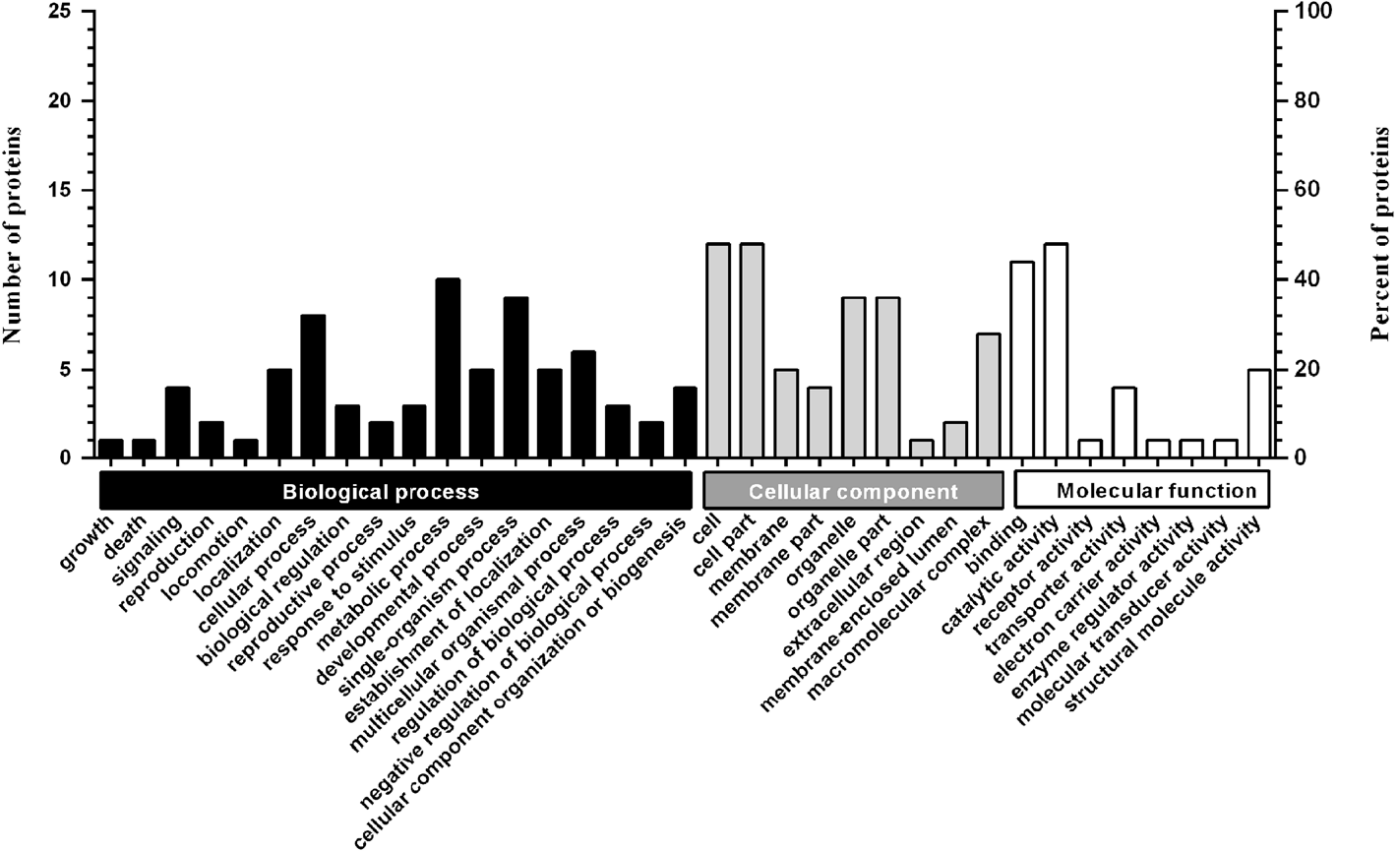
**Gene Ontology classification of differentially expressed proteins identified by iTRAQ experiments between the Lab-DS and Lab-DR strains.** The differentially expressed proteins are grouped into three hierarchically-structured GO terms: biological process, cellular component, and molecular function. The y-axis indicates the number and percent of proteins in each GO term.

Among the 30 differentially expressed proteins, 25 proteins were sub-categorized into 35 hierarchically-structured GO classifications including 18 biological process, 9 cellular component, and 8 molecular function (Figure 2). Specially, “metabolic process” (10, 40%), “single-organism process” (9, 36%), and “cellular process” (8, 32%) were highly represented in “biological process”; “cell” (12, 48%), “cell part” (12, 48%), and “organelle” (9, 36%) in “cellular component”; and “catalytic activity” (12, 48%), “binding” (11, 44%), and “structural molecule activity” (5, 20%) in “molecular function” (Figure 2, Additional file 5: Table S4).

### Validation of CYP6AA9 expression in the laboratory and field populations by Western blot

**Figure 3:**
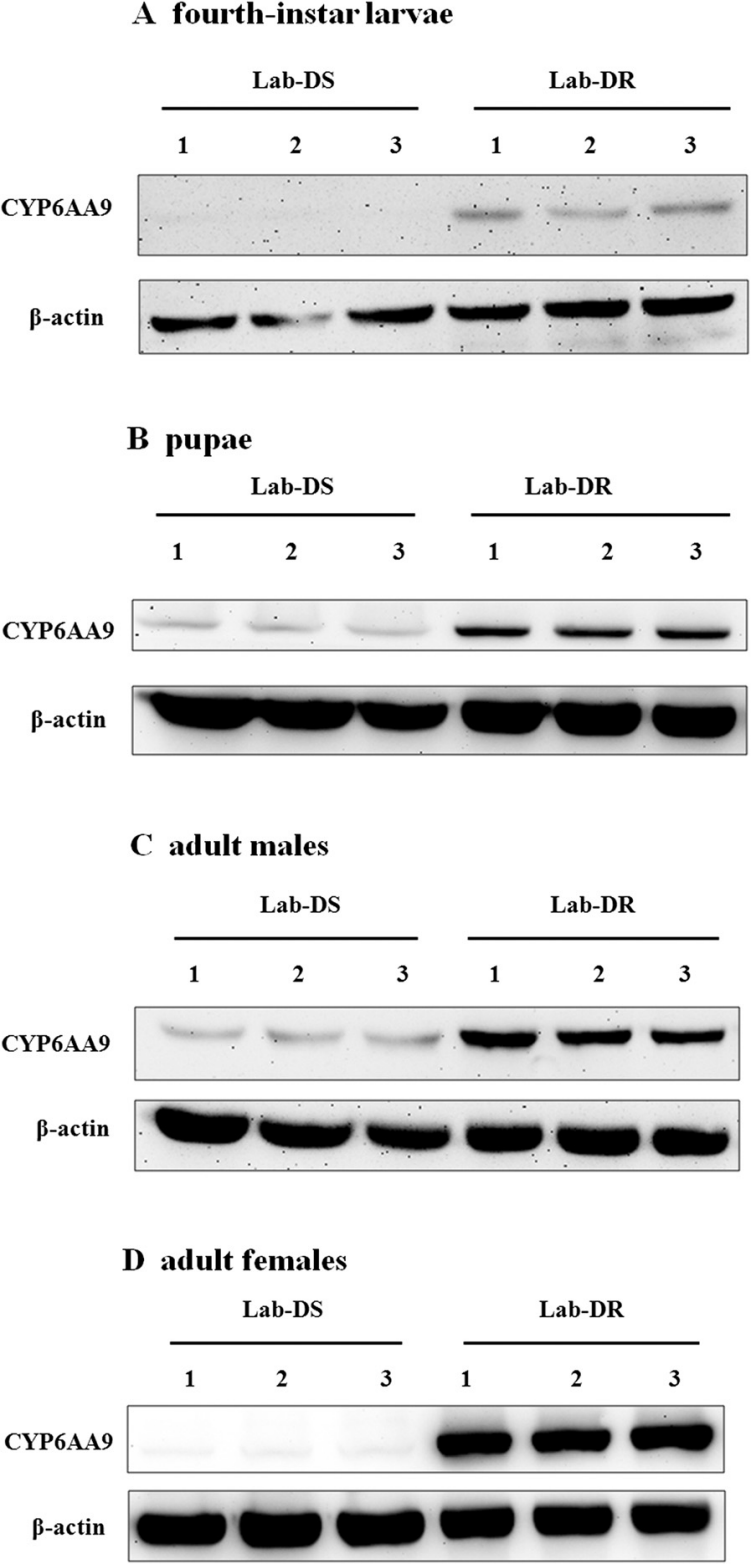
**Validation of CYP6AA9 expression at different life stages in the laboratory population by Western blot. (A)**, **(B)**, **(C)**, and **(D)** is indicated the fourth-instar larvae, pupae, adult males and adult females, respectively. β-actin was the internal control. Three biological replicates were performed for each life stage.

After preparing the antibody against CYP6AA9 successfully, we investigated the expression of CYP6AA9 at the four different life stages in the laboratory population by Western blot, which was conducted to validate the proteomic data. The results showed that CYP6AA9 was overexpressed at each life stage of the Lab-DR strain comparing to the corresponding life stage of the Lab-DS strain. At least three biological replicates were performed, and the representing results were shown in Figure 3.

**Figure 4:**
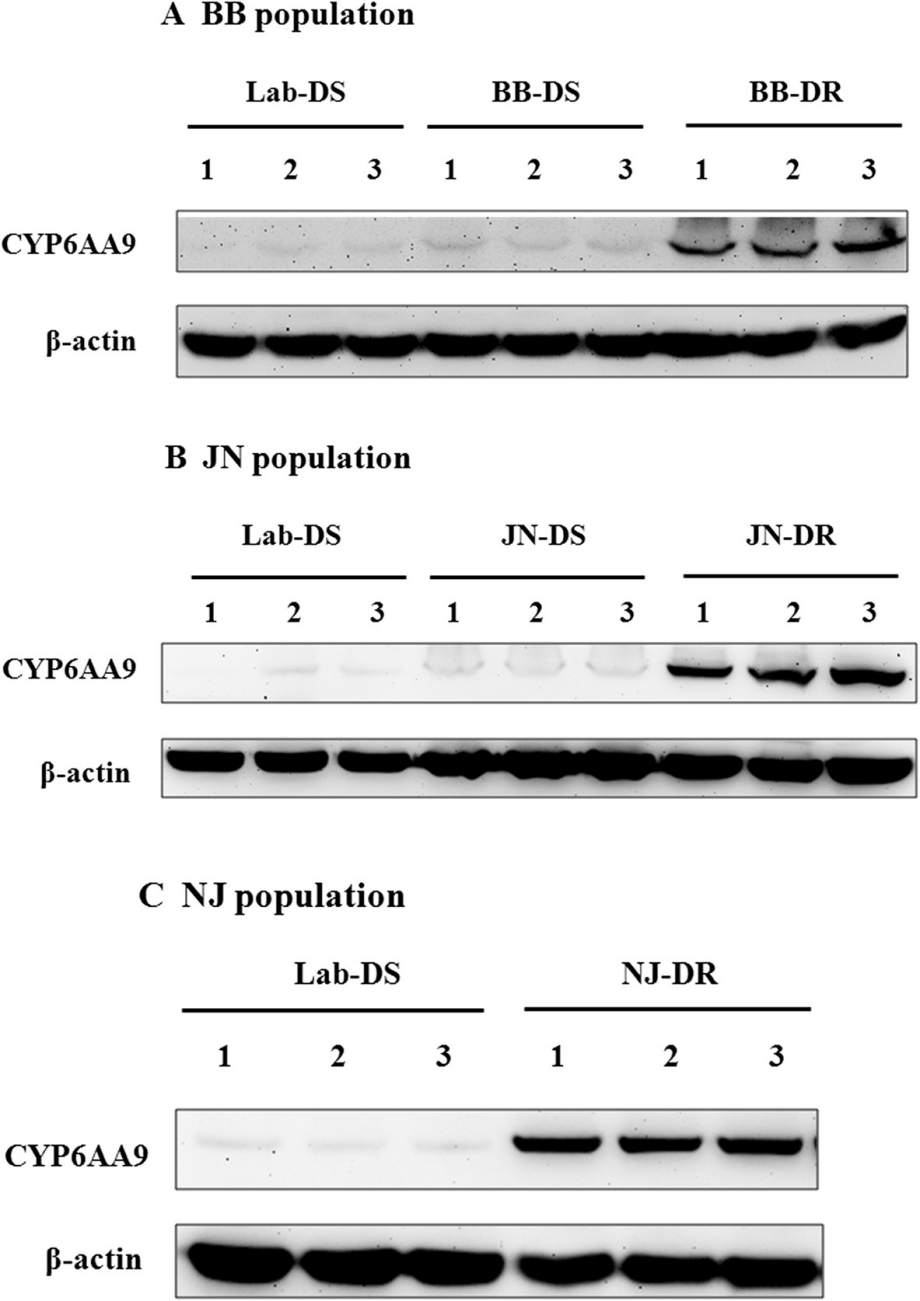
**Validation of CYP6AA9 expression in the three field populations by Western blot.** The Lab-DS strain was control, and β-actin was the internal control. **(A)**, **(B)**, and **(C)** is indicated the three field populations, respectively. Three biological replicates were performed for each field population. However, the expression of CYP6AA9 in the NJ-DS strain was not determined because of lack of samples for comparison.

Subsequently, we further investigated the expression of CYP6AA9 in BB, JN, and NJ populations by Western blot. After insecticide susceptibility tests, the knockdown rate of the three field populations was recorded, respectively (Additional file 6: Table S5), and the knockdown rate of NJ population was obviously the lowest. The Western blot results showed that CYP6AA9 was overexpressed in the DR strain of each field population comparing to the corresponding DS strain and/or Lab-DS strain (Figure 4). However, the expression of CYP6AA9 in the NJ-DS strain was not determined because of lack of enough samples for comparison.

## Discussion

Clarifying resistance mechanisms is essential for improving resistance management strategies. Despite recent progresses, molecular mechanisms underlying pyrethroid resistance remain to be poorly understood. Proteomics provide the feasibility to screen out pyrethroid resistance-related proteins at a large scale for further increasing our understanding of resistance mechanisms. However, there have been very few reports on the proteomics analysis of insecticide resistance in insect species so far. To the best of our knowledge, it is the first study to use modern proteomic tools for identifying pyrethroid resistance-related proteins in *Cx. pipiens*. The strengths of present study are to bring to light many proteins that were not previously thought to be associated with pyrethroid resistance. In total, there were 30 differentially expressed proteins identified in both replicate experiments. Based on function analysis, the differentially expressed proteins were then assigned into 10 different categories: oxidoreductase activity, transporter activity, transferase activity, catalytic activity, hydrolase activity, other enzymes, structural constituent of cuticle, nucleic acid binding, hypothetical proteins and others. It is indicated that more proteins may be involved in pyrethroid resistance beside some well-known ones such as P450s and GSTs, which further expands our understanding of pyrethroid resistance mechanisms.

It has been frequently proved that increased P450-mediated detoxification is a major mechanism of pyrethroid resistance in insects [40,41]. To date, more than 2,000 insect P450s have been assigned to 67 families based on the identity at the amino-acid sequence level [42,43], and the members of CYP6 family have been verified to be involved xenobiotic metabolism [44]. There are many reports demonstrating the relationship between pyrethroid resistance and elevated activity of CYP6 family members in different mosquito species [9]. For example, *CYP6Z1, CYP6Z2*, *CYP6M2* and *CYP6P3* gene were found overexpressed in pyrethroid-resistant strains of *An. gambiae* [45-50]; *CYP6M2* and *CYP6P3* in *An. arabiensis* [51]; *CYP6Z6*, *CYP6M6* and *CYP6M11* gene in *Ae. aegypti* [52,53]; *CYP6AA7* gene in *Cx. quinquefasciatus* [17,20,54]; and *CYP6F1* gene in *Cx. pipiens pallens* [55]. In the current study, CYP6AA9 identified overexpressed in the Lab-DR strain belongs to a member of CYP6 family and may be involved in pyrethroid resistance just like other members mentioned above. After obtaining the antibodies against CYP6AA9 successfully, we compared the expression of CYP6AA9 at different life stages (fourth-instar larvae, pupae, adult males and females) between the Lab-DS and Lab-DR strains by Western blot. The results showed that CYP6AA9 was overexpressed in the four life stages of the Lab-DR strain comparing with the Lab-DS strain, which was consistent with the proteomic data. Subsequent studies further showed the upregulation of CYP6AA9 in the DR strains of three field populations. It was reported recently that *CYP6AA9* gene was found transcriptionally overexpressed in both the fourth-instar larvae and adult females of pyrethroid-resistant strains of *Cx. quinquefasciatus* by quantitative real-time PCR (qRT-PCR) [54], then the results were further validated by RNA-seq [17]. In addition, its *An. gambiae* orthologue *CYP6AA1* gene was also revealed transcriptionally overexpressed in the adult females of pyrethroid-resistant strains by cDNA microarray [49], and its *Ae. aegypti* orthologue *CYP6AA5* gene was overexpressed in the fourth-instar larvae [52]. Taken together, such a strong association with pyrethroid resistance suggests that CYP6AA9 may play a key functional role in pyrethroid resistance. However, its potential to metabolize pyrethroids remains unknown. Confirmatory evidence of a direct role in pyrethroid resistance must come from in vitro metabolism of pyrethroids with heterologously expressed P450s [56]. Recently, limited members of CYP6 family, such as CYP6P3, CYP6M2 and CYP6P9b, were proved to directly metabolize pyrethroids [48,50,57,58]. The question whether CYP6AA9 has the capability to metabolize pyrethroids requires further investigations. More importantly, CYP6AA9 was found to be firmly associated with pyrethroid resistance in both the laboratory and field populations, which indicates that CYP6AA9 may be used as a potential marker to monitor and predict the pyrethroid resistance level of field mosquito populations.

In the results, there were five cuticle proteins identified differentially expressed in the Lab-DS and Lab-DR strains. Among them, cuticle protein CP14.6 precursor (VectorBase ID: CPIJ800196), cuticle protein (VectorBase ID: CPIJ001836) and pupal cuticle protein 78E (VectorBase ID: CPIJ012466) have been characterized as cuticle proteins clearly in the database of *Cx. quinquefasciatus*, and the others described as hypothetical proteins may also belong to cuticle proteins by protein BLAST the database of *An. gambiae*. One out of five cuticle proteins, cuticle protein CP14.6 precursor was found overexpressed in the Lab-DR strain. The overexpression of cuticle protein was considered to support the hypothesis that mosquitoes may protect themselves from insecticides by cuticular thickening or remodeling, which leads to the occurrence of cuticular resistance [47,59,60]. So far, some cuticle protein genes have been identified overexpressed in pyrethroid-resistant strains of several mosquito species, such as *CPLC8* gene in *An. stephensi* [61], *CPR30*, *CPLCG3* (formerly named *CPLC8* [62]) and *CPLCG4* (formerly named *CPLC#*) gene in *An. gambiae* [36,63,64] and *CPR125* gene in *An. funestus* [18]. Furthermore, CPLCG3 and CPLCG4 were found restrictedly in the endocuticle, which may contribute to the thickness of the cuticle [65]. Although cuticular resistance is frequently mentioned, the mechanism is less well understood. Recent studies reported that cuticular thickening was associated with pyrethroid resistance [59,66], which partially contributes to the explanation of cuticular resistance. In fact, the properties of insect cuticle including permeability to pyrethroids are influenced not only by the chitin sclerotization, construction, and hydration, but also by the regular combinations of different cuticular proteins and their arrangements [60,67]. Therefore, other alterations of cuticle structure and composition such as cuticular remodeling may be also involved in cuticular resistance. The question how cuticle proteins are involved in the process of cuticle alterations for slowing down the penetration of insecticides is puzzling, requiring further research.

ATP synthase B chain and G-subunit, described as two subunits of ATP synthase (also known as the F-ATPase or F_1_F_o_ ATP synthase) complex, were both identified overexpressed in the Lab-DR strain. For a long time, ATP synthase complex was considered to be present exclusively in mitochondrial membrane. Few years ago, some structural subunits of ATP synthase complex were detected on the cell surface of several tissues [68]. It is characterized that ATP synthase complex not only plays a central role in ATP production via H^+^ transport [69], but also participates in some processes, such as the regulation of apolipoprotein metabolism and cellular proliferation [68]. Recently, two genes coding for ATP synthase B chain and G-subunit were shown overexpressed in the *Bti*-resistant strain of *Ae. aegypti* [70]. There are no reports on the relationship between the two subunits and pyrethroid resistance so far. Based on their important role in ATP generation, upregulation of ATP synthase B chain and G-subunit might be beneficial to meet the requirement of energy during metabolic enzyme-mediated detoxification, which remains to be determined.

The gene *prag01* was previously characterized to be associated with deltamethrin resistance in our lab [71]. In present study, prag01 was shown overexpressed in the Lab-DR strain, further corroborating previous reports. However, the mechanism how prag01 is involved in pyrethroid resistance requires much more work to be done.

## Conclusions

It is the first study to identify pyrethroid resistance-related proteins using modern proteomic tools in *Cx. pipiens*. We found many proteins that were not previously thought to be associated with pyrethroid resistance, which further expands our understanding of pyrethroid resistance mechanisms. CYP6AA9 was overexpressed in the deltamethrin-resistant strains of the laboratory and field mosquito populations, indicating that CYP6AA9 may be involved in pyrethroid resistance and may be used as a potential genetic marker to monitor and predict the pyrethroid resistance level of field populations.

## Additional files

Additional file 1: Table S1. List of proteins identified and quantified by the iTRAQ analysis in the Rep1 and Rep2 experiments, respectively. Additional file 2: Table S2. List of up-regulated and down-regulated proteins identified and quantified by the iTRAQ analysis in the Rep1 experiment. Additional file 3: Table S3. List of up-regulated and down-regulated proteins identified and quantified by the iTRAQ analysis in the Rep2 experiment. Additional file 4: Figure S1. Representative MS/MS spectrum showing a peptide from CYP6AA9 (peptide sequence: DMTYLEQVVNESLR). Additional file 5: Table S4. Gene ontology analysis of differentially expressed proteins in *Cx. pipiens pallens* proteome. Additional file 6: Table S5. The knockdown rate of the three field populations of *Cx. pipiens pallens* after exposure to 0.05% deltamethrin for 80 minutes.
