# Supplementary figures and images for: Identification of proteins associated with pyrethroid resistance by iTRAQ-based quantitative proteomic analysis in Culex pipiens pallens
Source: Parasit Vectors. 2015 Feb 10;8:95. doi: 10.1186/s13071-015-0709-5 (PMC4337324; doi:10.1186/s13071-015-0709-5)

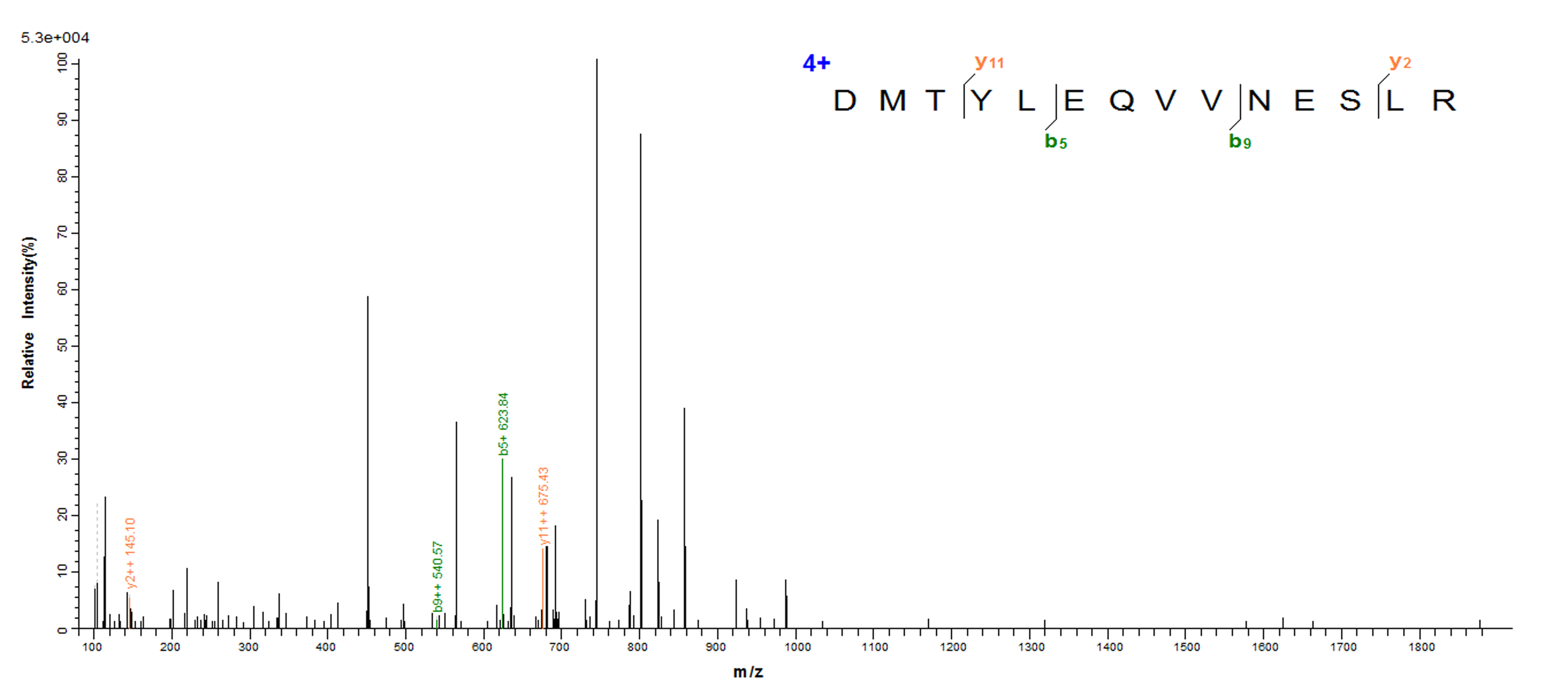

Supplement: Additional file 4: Figure S1. — Representative MS/MS spectrum showing a peptide from CYP6AA9 (peptide sequence: DMTYLEQVVNESLR). [file 13071_2015_709_MOESM4_ESM.tiff]
