# Supplementary material for: Identification of proteins associated with pyrethroid resistance by iTRAQ-based quantitative proteomic analysis in Culex pipiens pallens
Source: Parasit Vectors. 2015 Feb 10;8:95. doi: 10.1186/s13071-015-0709-5 (PMC4337324; doi:10.1186/s13071-015-0709-5)
Supplement: Additional file 6: Table S5. — The knockdown rate of the three field populations of Cx. pipiens pallens after exposure to 0.05% deltamethrin for 80 minutes. [file 13071_2015_709_MOESM6_ESM.doc]

Table S5. The knockdown rate of the three field populations of *Cx. pipiens pallens* after exposure to 0.05% deltamethrin for 80 mins.

| **Field population** | **Total no. exposed** | **Knock down no.** | **Knock down rate（%）** |
| --- | --- | --- | --- |
| BB | 446 | 74 | 16.6 |
| JN | 592 | 70 | 11.8 |
| NJ | 91 | 3 | 3.3 |
